# The incidence and influencing factors of postoperative acute kidney injury in elderly patients with hip fractures: A meta-analyses

**DOI:** 10.1371/journal.pone.0322228

**Published:** 2025-06-20

**Authors:** Chong Hou, Peizhe Zhang, Yuan Xue, Jinli Guo

**Affiliations:** 1 Shanxi Medical University, Taiyuan, China; 2 The Second Hospital of Shanxi Medical University, Taiyuan, China; Tehran University of Medical Sciences, IRAN, ISLAMIC REPUBLIC OF

## Abstract

**Objective:**

Acute kidney injury (AKI) is one of the common complications after hip fracture in the elderly, and its hospitalization rate, mortality rate and medical expenses are high, resulting in serious social and economic burden. Therefore, we aim to systematically evaluate the incidence and risk factors of postoperative AKI in elderly patients with hip fracture, so as to identify the occurrence of postoperative AKI in elderly patients with hip fracture early, so as to prevent it in advance.

**Methods:**

This meta-analysis adhered to PRISMA guidelines and was registered in PROSPERO (CRD42024498009).We systematically searched Embase, Pubmed, web of science, the cochrane Library, CBM, VIP, CNKI, and Wanfang Data to collect studies on the incidence or influencing factors of postoperative acute kidney injury in elderly patients with hip fracture published up to 18 December 2023. All studies were screened, relevant data extracted, and quality assessed by two independent authors, and meta-analysis was performed using Stata 15.0 software.

**Results:**

A total of 22 articles were included, with a total sample size of 25195 cases and 16 influencing factors. The results of meta-analysis showed that the incidence of postoperative AKI in elderly patients with hip fracture was 17.2% [95%CI (14.3% ~ 20%), P < 0.0001], and there were 6 statistically significant influencing factors, which were baseline serum potassium [OR(95%CI)=2.23 (1.22, 4.05)], hypertension [OR(95%CI)=3.00 (1.75, 5.88)], and chronic kidney disease [OR(95%CI)=4.40 (1.10, 14.75)], diabetes mellitus [OR (95% CI) = 1.84 (1.19, 2.83)], intraoperative hypotension [OR (95% CI) = 5.61 (3.36, 9.35)], and operative time [OR (95% CI) = 1.01 (1.00, 1.02)].

**Conclusions:**

Our study indicated that the incidence of postoperative AKI in elderly patients with hip fracture was 17.2%. Baseline serum potassium, hypertension, chronic kidney disease, diabetes, operative hypotension and operation time were the influencing factors of postoperative AKI in elderly patients with hip fracture.

PROSPERO registration number for this study: CRD42024498009

## Introduction

With the advancement of medical level, the life expectancy of human beings increases, and the problem of population aging also follows [1]. Elderly osteoporotic fractures are also increasing year by year, especially hip fractures, which have serious postoperative complications, high disability rate, mortality rate and medical costs, and have become an important public health problem threatening the life and health of elderly people. According to epidemiological studies, the proportion of hip fractures in people over the age of 80 has increased by 7% [2], and the number of related operations is expected to continue to rise by 2% per year over the next 30 years [3]. In this context, postoperative acute kidney injury (AKI), as a serious complication after hip fracture, is becoming more and more urgent. Clinical evidence shows that the incidence of AKI after hip fracture in the elderly is as high as 28.4% [4]. This complication significantly increased the postoperative mortality, prolonged hospital stay and increased the cost of medical resource utilization [5–7]. It is worth noting that the unique physiological characteristics of elderly patients (such as decreased kidney reserve function, decreased vascular elasticity) and surgical stress have a superposition effect, making AKI prevention more challenging. Therefore, early identification of influencing factors is essential to reduce postoperative AKI in elderly patients with hip fractures. There are many factors for AKI after hip fracture, including age, sex, body mass index (BMI), diabetes, chronic kidney disease (CKD), intraoperative hypotension, Charlson comorbidity index (CCI), anesthesia, and glomerular filtration rate (eGFR) [8–10]. Despite some progress in previous studies, the available findings lack consistency and evidence-based research remains limited.

In 2021, a meta-analysis of Li [11] studied the incidence rate and risk factors of postoperative AKI in patients with hip fracture, but the study had some limitations. The heterogeneity of incidence rate was high, and the source of heterogeneity could not be found. In addition, because there were few included studies, the research results only pointed out that postoperative albumin was a risk factor of postoperative AKI in patients with hip fracture. In addition to the above issues, the emergence of new evidence and different incidence reports in recent years have also become an obstacle to providing clear indicators or recommendations for postoperative acute kidney injury in elderly patients with hip fractures. There is therefore an urgent need to integrate the latest evidence through systematic analysis to clarify what is happening and the factors involved. In summary, this study aims to clarify the incidence and influencing factors of AKI in elderly patients undergoing hip fracture surgery through meta-analysis, and provide scientific basis for early reduction and prevention of AKI in clinical practice.

## Materials and methods

This meta-analysis was undertaken according to the guideline of the Meta-analysis of Observational Studies in Epidemiology (MOOSE) checklist and the Preferred Reporting Items for Systematic Reviews and Meta-Analysis (PRISMA) statement. (Table S1)

### Literature search

Retrieve relevant content on “Factors Influencing Postoperative AKI of Hip Fracture” on Chinese and English search websites, using a combination of theme words and free words, supplemented by manual retrieval to begin the search. The search time range is from database establishment to December 18, 2023. The retrieved databases include Pubmed, Web of Science, Embase, Cochrane Library, CNKI, Wanfang Database, CBM, and VIP Database. The literature search terms are [hip fracture, intertrochanteric fracture, subtrochanteric fracture, femoral neck fracture, hip joint fracture, subtrochanteric fracture, or intertrochanteric fracture] AND [renal failure, renal injury, AKI, renal insufficiency, renal injury, or renal injury] AND [predictive factor, influencing factor, influencing factor, or related factor]. **(See** S2 Table **for details)**

### Study selection

Inclusion criteria: (1) Study type: case-control study, cohort study, cross-sectional survey, etc. (2) Research subject: Age over 65 years old, undergoing surgical treatment, confirmed as AKI through symptoms, signs, and laboratory tests. Exclusion criteria: (1) Patients diagnosed with AKI prior to this fracture. (2) Review or conference paper. (3) Repetitive literature. (4) Literature with incomplete data. (5) The method quality score is less than 5 points. Outcome measures: Whether patients with hip fractures have postoperative AKI and the influencing factors of AKI.

### Data extraction

All data were extracted from all eligible studies by two authors (Chong Hou and Peizhe Zhang). Extract the following variables from each study: (1) first author and publication year of the article, (2)basic characteristics of literature: research type, research duration, research area, research time, diagnostic criteria, total sample size, number of AKI, number of people without AKI, incidence rate of AKI and influencing factors. Any differences are resolved through discussion or consultation with senior reviewers to reach consensus. **(See** Tables 1 **and** S1 **for details)**

**Table 1:** Basic characteristics and quality evaluation table of the included literature.

Inclusion in research	Year	Research type	sample size	country	incidence rate	diagnosis standard	NOS score	influence factor
**Yuan** [8]	**2023**	**Retrospective research**	**307 (33/274)**	**China**	**0.1075**	**KDIGO**	**7**	**CCI ≥ 2, intraoperative hypotension**
**Wang** [14]	**2023**	**Retrospective research**	**644 (78/566)**	**China**	**0.121**	**KDIGO**	**9**	**Underweight**
**Pan** [15]	**2023**	**Retrospective cohort study**	**448 (60/388)**	**China**	**0.133**	**KDIGO**	**9**	**Age, CKD, ASA, bone cement, decreased hemoglobin**
**Zhan** [16]	**2022**	**Retrospective case-control study**	**308 (37/271)**	**China**	**0.12**	**KDIGO**	**8**	**Postoperative albumin, intraoperative hypotension, changes in hemoglobin**
**Agar** [9]	**2022**	**Retrospective research**	**589 (58/531)**	**Türkiye**	**0.098**	**KDIGO**	**8**	**Postoperative albumin, preoperative urea, postoperative urea, postoperative eGFR, postoperative serum potassium**
**Zhang** [10]	**2021**	**Retrospective cohort study**	**470 (194/276)**	**China**	**0.413**	**KDIGO**	**7**	**Diabetes, coronary heart disease, hypotension, WBC, creatinine, BUN, ALB, baseline eGFR, length of stay**
**Christensen** [4]	**2021**	**Retrospective cohort study**	**299 (85/214)**	**Denmark**	**0.284**	**KDIGO**	**7**	**_________**
**Kang** [17]	**2020**	**Retrospective case-control study**	**550 (25/525)**	**Korea**	**0.044**	**AKIN**	**8**	**Hospitalization time, postoperative albumin, estimated blood loss**
**Rantalaiho** [5]	**2019**	**Prospective cohort study**	**475 (40/435)**	**Finland**	**0.084**	**KDIGO**	**7**	**Blood creatinine, dementia**
**Jang** [18]	**2019**	**Retrospective research**	**248 (44/204)**	**Korea**	**0.177**	**KDIGO**	**9**	**Intraoperative hypotension**
**Cho** [19]	**2019**	**Retrospective cohort study**	**285 (67/218)**	**Korea**	**0.235**	**KDIGO**	**8**	**Hypertension, E/e ‘ratio, hemoglobin**
**Porter** [20]	**2017**	**cohort study**	**2848 (683/2165)**	**Britain**	**0.24**	**KDIGO**	**8**	**Age, male, CKD, two or more comorbidities**
**Hong** [7]	**2017**	**Retrospective cohort study**	**450 (95/355)**	**Korea**	**0.211**	**AKIN**	**7**	**_________**
**Marty** [21]	**2016**	**Prospective observational research**	**48 (29/19)**	**France**	**0.604**	**AKIN**	**8**	**Baseline glomerular filtration rate, postoperative RI**
**Ulucay** [22]	**2012**	**Prospective cohort study**	**163 (25/138)**	**Türkiye**	**0.153**	**AKIN**	**8**	**Female, baseline eGFR**
**Eren** [23]	**2012**	**Historical queue research**	**214 (36/178)**	**Türkiye**	**0.168**	**RIFLE**	**8**	**Female, baseline eGFR, serum potassium**
**Moon** [24]	**2022**	**Retrospective cohort study**	**307 (33/274)**	**Korea**	**0.105**	**AKIN**	**8**	**Age, male, intraoperative hypotension, CKD, use of ACEI, ALP**
**AbuSaleh** [25]	**2023**	**Retrospective cohort study**	**250 (29/221)**	**Israel**	**0.116**	**KDIGO**	**7**	**Baseline glomerular filtration rate, catheter**
**Ganta** [26]	**2021**	**Retrospective cohort study**	**1898 (149/1749)**	**United States**	**0.079**	**KDIGO**	**8**	**Female, CCI index, use of anticoagulants or platelet drugs, semi hip replacement surgery**
**Pedersen** [27]	**2017**	**Regional queue research**	**13529 (1717/11812]**	**Denmark**	**0.127**	**KDIGO**	**8**	**Obesity**
**Rutenberg** [28]	**2019**	**Retrospective cohort study**	**217 (55/162]**	**Israel**	**0.25**	**AKIN**	**7**	**_________**
**Küpeli** [29]	**2020**	**Retrospective research**	**160 (28/132]**	**Türkiye**	**0.175**	**KDIGO**	**6**	**_________**

Note: CCI: Chalson’s Comorbidity Index ASA: American Society of Anesthesiologists Score WBC: White blood cell BUN: Blood urea nitrogen CKD: Chronic kidney disease ALB: Albumin ALP: Alanine transaminase RI: Doppler renal resistance index E/e ‘: Ratio of mitral valve velocity to early diastolic velocity of mitral valve.

### Quality assessment

Two independent researchers conducted literature screening and quality evaluation. If there are any differences, they should be discussed with a third researcher. The Newcastle Ottawa Scale (NOS) was used for quality evaluation, with a score of ≤ 5 for low-quality studies, ≤ 6 for medium quality studies, and ≤ 7 for high-quality studies [11]. **(The evaluation information is shown in** Table 2)

**Table 2:** NOS scoring system for quality assessment.

Literature	Study population selection	Entry 1	Entry 2	Entry 3	Entry 4	Inter-group comparability	Result Measurement	Entry 1	Entry 2	Entry 3	Total points
**Linli Zhang**	**3**	**1**	**1**	**0**	**1**	**2**	**2**	**1**	**0**	**1**	**7**
**Xin Yuan**	**3**	**1**	**1**	**0**	**1**	**2**	**2**	**1**	**0**	**1**	**7**
**Hao Wang**	**4**	**1**	**1**	**1**	**1**	**2**	**3**	**1**	**1**	**1**	**9**
**Liping Pan**	**4**	**1**	**1**	**1**	**1**	**2**	**3**	**1**	**1**	**1**	**9**
**Sizheng Zhan**	**4**	**1**	**1**	**1**	**1**	**2**	**3**	**1**	**0**	**1**	**8**
**Abhishek Ganta**	**4**	**1**	**1**	**1**	**1**	**2**	**2**	**1**	**0**	**1**	**8**
Anil Agar	**4**	**1**	**1**	**1**	**1**	**2**	**3**	**1**	**0**	**1**	**8**
**Cagatay Ulucay**	**4**	**1**	**1**	**1**	**1**	**2**	**2**	**1**	**0**	**1**	**8**
**Philippe Marty**	**3**	**1**	**1**	**0**	**1**	**2**	**3**	**1**	**1**	**1**	**8**
**A B Pedersen**	**3**	**1**	**1**	**0**	**1**	**2**	**3**	**1**	**1**	**1**	**8**
**Christine J Porter**	**3**	**1**	**1**	**0**	**1**	**2**	**3**	**1**	**1**	**1**	**8**
**Woo Young Jang**	**4**	**1**	**1**	**1**	**1**	**2**	**3**	**1**	**1**	**1**	**9**
**Ida Rantalaiho**	**2**	**0**	**1**	**0**	**1**	**2**	**3**	**1**	**1**	**1**	**7**
**Joon Soon Kang**	**4**	**1**	**1**	**1**	**1**	**2**	**2**	**1**	**0**	**1**	**8**
**Julie Braüner Christensen**	**3**	**1**	**1**	**0**	**1**	**2**	**2**	**1**	**0**	**1**	**7**
**Seong Eun Hong**	**2**	**0**	**1**	**0**	**1**	**2**	**3**	**1**	**1**	**1**	**7**
**Tal Frenkel Rutenberg**	**3**	**1**	**1**	**0**	**1**	**2**	**2**	**1**	**0**	**1**	**7**
**İlke Küpeli**	**2**	**0**	**1**	**0**	**1**	**2**	**2**	**1**	**0**	**1**	**6**
**Woori Cho**	**3**	**1**	**1**	**0**	**1**	**2**	**3**	**1**	**1**	**1**	**8**
**Z. Eren**	**3**	**1**	**1**	**0**	**1**	**2**	**3**	**1**	**1**	**1**	**8**
**jun-kiMoon**	**3**	**1**	**1**	**0**	**1**	**2**	**3**	**1**	**1**	**1**	**8**
**Alaa Abu-Saleh**	**3**	**0**	**1**	**0**	**1**	**2**	**2**	**1**	**0**	**1**	**7**

### Data synthesis and analysis

Statistical analysis was performed using Stata 15.0 software, and the combined effect size 0R and 95% CI were calculated. If the Z-test result is P < 0.05, the difference is considered statistically significant. The P-value of I^2^ combined with X^2^ test was used to evaluate heterogeneity. Due to the inherent variability of observational studies, heterogeneity is usually high, due to differences in study design, sample source, and other factors. Therefore, a higher I2 threshold of 60%−75% is generally considered acceptable [12]. When there is clear reason to believe that the relative treatment effect is common across all included studies, the fixed-effect model is selected. When the expected therapeutic effects are similar but not identical, a random effects model is chosen [13]. In this study, when the I2 value is less than 60%, it is considered that there is little heterogeneity, indicating that the effect size of each study is similar, so the common effect can be assumed and the fixed effect model can be selected. Otherwise, the random effects model is chosen. When the heterogeneity was significant and the number of studies was ≥ 20, meta-regression analysis was performed according to diagnostic criteria, study type, sample size, study area, and study duration to explore the source of heterogeneity.Sensitivity analyses were performed for included studies to assess the stability of the results by excluding individual studies or switching analysis models. Egger test was used to evaluate the potential publication bias of literature, and P < 0.05 was considered statistically significant.

## Results

### Literature search results

**Fig 1:**
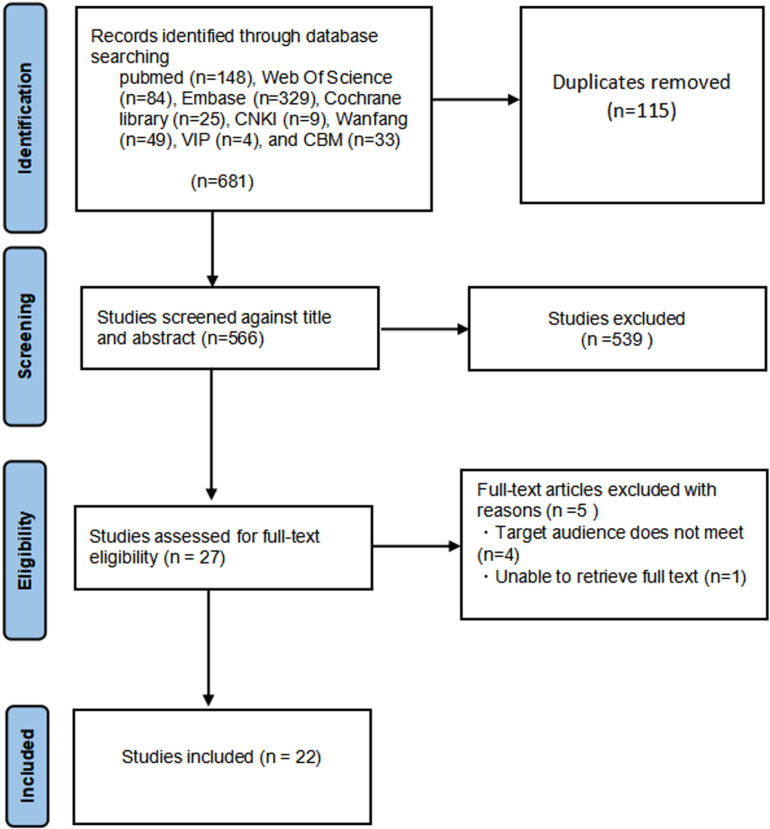
Flow chart of literature screening.

Through preliminary literature search, a total of 681 articles were obtained, with 115 duplicate articles removed. 27 articles were selected based on reading titles and abstracts, and 22 articles were selected according to inclusion and exclusion criteria. Among them, 2 are in Chinese and 20 are in English. The literature screening process is shown in Fig 1.

### Basic characteristics of included literature

A total of 22 articles were included, including 19 retrospective studies and 3 prospective studies. A total of 25195 cases were included, including 3650 AKI patients and 21545 non AKI patients. The publication time of the literature is from the establishment of the database to December 18, 2023. The basic characteristics of the literature are shown in Table 1.

### Meta analysis results

**Fig 2:**
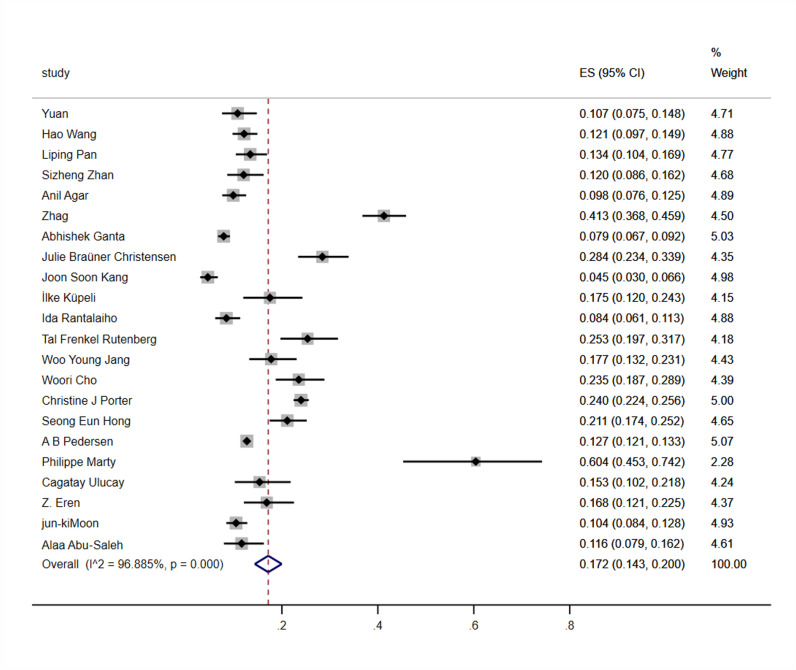
Forest plot of postoperative AKI incidence.

**Fig 3:**
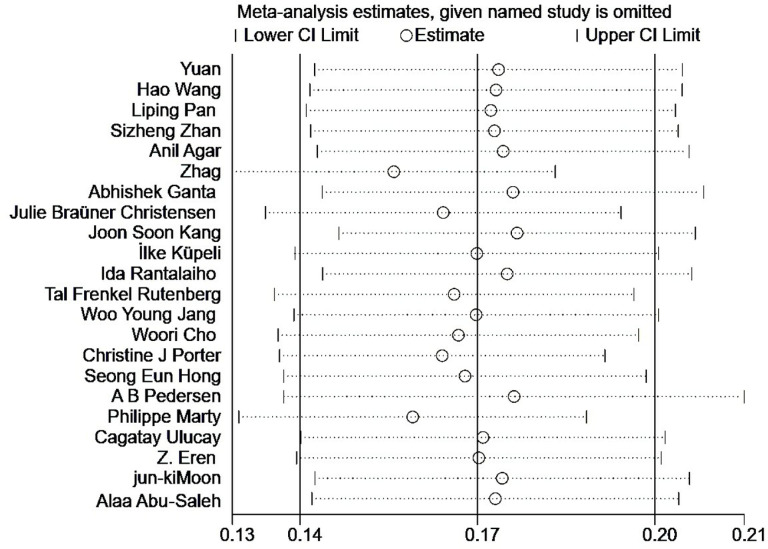
Sensitivity analysis of postoperative AKI incidence.

22 articles were subjected to heterogeneity testing, and the results showed significant heterogeneity, with ^*I2*^ = 96.9% and *P* < 0.001. The random effects model was used for meta-analysis.The results showed that the incidence rate of acute renal injury after hip fracture surgery was 17.2% [95% *CI* (14.3%, 20.0%)] (Fig 2). Using metaregression to analyze the sources of heterogeneity, based on diagnostic criteria, study type, sample size, study region, and study duration, the results showed that study duration (*P* = 0.012) and sample size (*P = 0.049)* may be the sources of heterogeneity in the occurrence of AKI. After conducting sensitivity analysis and removing any literature one by one, the results did not show significant changes, indicating stable and reliable results (Fig 3). The Egger test was used to determine publication bias, and the results showed no statistically significant difference (*t* = 1.72, *P* = 0.101), indica*t*ing a lower possibility of publication bias.

### Factors influencing postoperative AKI

Heterogeneity test was performed for 16 influencing factors included in the study. The results showed that the random-effects model was used for meta-analysis of the included literatures in terms of age, male, female, BUN, length of stay, preoperative albumin, postoperative serum albumin, preoperative serum creatinine, chronic kidney disease, CCI index, and baseline glomerular filtration rate. A fixed effect model was used for meta-analysis of baseline potassium, intraoperative hypotension, diabetes, operative time, and hypertension in the literature. Meta-analysis showed that baseline potassium, chronic kidney disease, diabetes, hypertension, intraoperative hypotension, and operative time were the influencing factors for postoperative AKI in elderly patients with hip fracture (P < 0.05). Age, male, female, BUN, baseline glomerular filtration rate, preoperative albumin, preoperative creatinine, postoperative serum albumin, CCI index, and length of stay were not influencing factors for postoperative AKI in elderly patients with hip fracture (P < 0.05). The sensitivity analysis of the results was carried out, and the combined effect size was calculated by changing the two effect models. The results show that the combined effect size under the two models does not change significantly, indicating that the results are stable and reliable. (Table 3)

**Table 3:** Meta analysis and sensitivity analysis of risk factors for AKI after hip fracture surgery.

influence factor	Inclusion in research	*I* ^*2*^	P1	*OR* (95% CI)		P2
				**Fixed effects model**	**Random effects model**	
**Age**	**9**	**88.2**	**0.000**	**1.03 [1.01,1.04]**	**1.05 [1.00, 1.11]**	**0.051**
**masculine**	**2**	**80.3**	**0.024**	**1.54 [1.27,1.88]**	**2.45 [0.75,8.06]**	**0.14**
**woman**	**4**	**63.6**	**0.041**	**0.67 [0.47, 0.96]**	**0.76 [039,1.48]**	**0.412**
**Diabetes**	**4**	**0**	**0.42**	**1.84 [1.19,2.83]**	**1.84 [1.19, 2.83]**	**0.006**
**BUN**	**3**	**85.5**	**0.001**	**1.03 [0.99,1.07]**	**1.12 [0.89, 1.42]**	**0.335**
**Hospitalization time**	**2**	**91.6**	**0.00**	**1.44 [1.14, 1.81]**	**2.22 [0.66, 7.52]**	**0.199**
**Baseline glomerular filtration rate**	**7**	**89.6**	**0.000**	**1.00 [1.00, 1.01]**	**0.98 [0.95, 1.01]**	**0.154**
**Baseline serum potassium**	**2**	**0**	**0.422**	**2.23 [1.22, 4.05]**	**2.23 [1.22, 4.05]**	**0.008**
**Postoperative serum albumin**	**3**	**95.5**	**0.00**	**1.27 [1.00, 1.62]**	**0.40 [0.1, 1.65]**	**0.16**
**Preoperative blood creatinine level**	**3**	**88.1**	**0.000**	**1.01 [1.01,1.01]**	**1.01 [0.99,1.02]**	**0.53**
**Intraoperative hypotension**	**3**	**0**	**0.866**	**5.61 [3.36,9.35]**	**5.61 [3.36, 9.35]**	**0.000**
**CCI index**	**2**	**70.9**	**0.064**	**1.22 [1.10,1.36]**	**1.72 [0.68,4.33]**	**0.25**
**Preoperative serum albumin**	**3**	**82.1**	**0.004**	**1.00 [0.90,111]**	**1.38 [0.83,2.30]**	**0.22**
**Surgical time**	**2**	**32.8**	**0.223**	**1.01 [1.00,1.02]**	**1.01 [1.00,1.03]**	**0.022**
**Chronic kidney disease**	**4**	**89.7**	**0.00**	**1.85 [1.47,2.32]**	**4.40 [1.10,14.75]**	**0.035**
**hypertension**	**2**	**5.6**	**0.303**	**3.00 [1.57, 5.73]**	**3.01 [1.55,5.88]**	**0.001**

Note: P1 represents the result of heterogeneity testing; P2 is the result of selecting the effect model based on the value of z and conducting a z-test.

## Discussion

Among the 22 studies included with good methodological quality, 16 of them were published within the past five years (2019–2023), and the NOS scores of the included studies were 6–8 points, indicating that the literature quality was above average. Therefore, the overall conclusions of the studies are relatively reliable. The statistical heterogeneity of this study is mainly related to factors such as different inclusion and exclusion criteria, differences in sample size, and study regions.

### The incidence of AKI after hip fracture surgery is high

This study conducted a meta-analysis of the incidence of AKI after hip fracture surgery in elderly patients. The results showed that the incidence of AKI was 17.2%. This is consistent with the incidence of AKI in elderly hip fractures studied previously, which was 11.8–24% [24]. The incidence rate is 10.75% higher than that of Yuan et al. [8], and 41.3% lower than that of Zhang et al. [10], which may be related to differences in the included population and research period. The incidence rate of AKI in elderly patients after hip fracture surgery is high. The study found that the risk of AKI in patients aged 70 to 79 years after hip replacement is 5.196 times that in patients under 50 years of age, and 11.227 times that in patients over 80 years of age [30]. One possible reason is that elderly patients have poor tolerance to surgical anesthesia, which can lead to severe hemodynamic fluctuations during surgery, resulting in insufficient renal blood flow perfusion and an increased risk of AKI; On the other hand, the organ function of elderly patients gradually deteriorates, with an increase in underlying diseases, a decrease in renal physiological reserve function, capacity depletion, susceptibility to infection, and exposure to nephrotoxins, further exacerbating the occurrence of postoperative acute kidney injury [28,31-32], which is consistent with the research results of Li Chen et al. [33]. Therefore, medical personnel should pay attention to the influencing factors of postoperative AKI in elderly patients with hip fractures, adopt active and effective strategies, and reduce the occurrence of postoperative AKI.

### Factors influencing the occurrence of AKI in elderly patients with hip fractures

#### General factors affecting postoperative AKI in elderly patients with hip fractures.

The results of this study indicate that baseline serum potassium is a risk factor for postoperative AKI in elderly patients with hip fractures. The serum potassium in the human body is mainly excreted by the kidneys to maintain the balance of intracellular and extracellular potassium ion concentrations. When serum potassium is too high or too low, it can cause serious harm to the body. If the potassium concentration in the blood is too low, it may cause damage to the renal tubules, resulting in vacuolar degeneration of the renal tubules, which in turn affects their function and impairs their ability to concentrate urine. If hypokalemia persists and blood urea and creatinine levels increase, it further exacerbates renal damage; Excessive blood potassium concentration can also cause certain harm to the kidneys, especially for CKD patients, which is consistent with the research of Thongprayoon and Lombardi et al. [34,35]. In the study by Chen et al. [36], it was also pointed out that patients with serum potassium>4.6 mmol/L have a higher risk of progressing to AKI stage 3 than patients with serum potassium ≤ 4.6 mmol/L. Therefore, during the period of fluid replacement for patients with fractures, it is necessary to monitor the patient’s serum potassium and other related electrolytes in a timely manner, control blood potassium levels, and prevent low or high blood potassium levels, which is essential for preventing postoperative AKI.

#### Chronic disease factors affecting postoperative AKI in elderly patients with hip fractures.

The results of this study show that hypertension, CKD and diabetes are the influencing factors of AKI after hip fracture surgery in the elderly. Patients with concomitant hypertension may experience renal arteriosclerosis and even renal parenchymal damage after fractures due to their own vascular lesions and hemodynamic changes, affecting the normal blood pressure circadian rhythm. Continuous high perfusion can also damage the glomerular filtration membrane and renal tubules [37], exacerbating renal damage, which is consistent with the findings of Yanay et al. [38]. Research has shown [39] that controlling blood pressure levels is a protective factor for renal dysfunction in hypertensive individuals. Therefore, strict control of blood pressure in clinical practice is of great significance in preventing renal dysfunction in hypertensive individuals and delaying the progression of kidney disease.

Combined CKD is a risk factor for postoperative AKI in elderly patients with hip fractures. Research has shown that if there is stage 3 CKD before surgery, the risk of developing AKI after surgery will be four times higher than that of the general population [30]. The reason may be that the kidney structure and function of CKD patients are damaged, which weakens their kidney defense ability; Secondly, CKD patients have a decrease in their own immune function and are prone to infection, leading to kidney damage and systemic inflammatory reactions, further deteriorating kidney function [40]; The use of contrast agents, drugs, and insufficient renal perfusion can also lead to kidney damage, which is consistent with the findings of Shin et al. [41].

The risk of AKI in diabetes patients after surgery will be significantly increased, 2.377 times higher than that in non diabetes patients [42,43]. The possible reason is that the microvascular disease caused by diabetes is an important mechanism leading to damage to renal function, and hyperglycemia leads to abnormal renal hemodynamics and tissue secretion and metabolism, resulting in diabetes nephropathy. Compared with normal people, diabetes has decreased renal oxygenation and vascular damage, which increases the possibility of complicated infections. Heart failure and septic shock caused by severe infection will lead to insufficient renal blood perfusion, further aggravate renal damage, and cause AKI [44]. Therefore, for patients with diabetes, blood sugar should be monitored dynamically in real time, and attention should be paid to the control and maintenance of blood sugar. When blood sugar is too high, timely measures should be taken to remedy it to prevent the occurrence and development of kidney injury.

#### Intraoperative factors affecting postoperative AKI occurrence.

This study shows that surgical time and intraoperative hypotension are influencing factors for postoperative AKI in elderly patients with hip fractures. Hip fracture surgery is usually more complex [45,46], involving multiple parts such as the hip joint and femoral neck. For elderly patients with other diseases, prolonged surgery time may have adverse consequences after surgery. Studies have shown that surgery time > 2 hours increases the risk of postoperative AKI [47], and surgery time is more than 12 hours, which increases the risk of death [48]. However, there is currently limited research on the impact of surgical duration on the occurrence of postoperative AKI, and there is no clear time frame to predict its occurrence. Therefore, in clinical practice, surgical time should be strictly controlled, surgical procedures should be followed, and unexpected situations such as excessive bleeding and coagulation dysfunction should be avoided to prevent the extension of surgical time.

When there is significant bleeding from a hip fracture, the peripheral blood volume decreases and the average arterial pressure decreases. When the average arterial pressure continues to be below 65 mm Hg, the self-regulation function of the kidneys will be basically lost, leading to insufficient renal perfusion and postoperative AKI [34]. There are various factors that can cause intraoperative hypotension. In addition to blood loss, surgical anesthesia, fluid replacement, and surgical position are also important factors [20]. Research has found that even a short duration of intraoperative hypotension (mean arterial pressure<55 mm Hg for 10 minutes or mean arterial pressure<60 mm Hg for 20 minutes) may lead to the occurrence of perioperative AKI in non cardiac surgery [49]. Therefore, when planning surgery for elderly patients with hip fractures, attention should be paid to the risk of AKI caused by intraoperative hypotension. Strict intraoperative blood pressure management will be a very beneficial choice for preventing postoperative AKI.

Given these results, the incidence of AKI after hip fracture in older patients is not low (17.2%), which requires concerted efforts to reduce the incidence of acute kidney injury (AKI) after hip fracture in older patients. It emphasizes the need for a multifaceted approach to reduce risk, with a focus on older people with underlying conditions [50]. For intraoperative hypotension and operation time, although it cannot be accurately predicted before surgery, real-time intraoperative monitoring and recording of these data can help doctors identify patients at increased risk of postoperative AKI in a timely manner. In addition, the impact of each risk factor varies in different patients, and considering these factors together helps to develop a personalized risk assessment plan for patients [15,51].

In the formulation of preventive measures, for basic diseases such as hypertension, chronic kidney disease and diabetes, the condition can be actively controlled before surgery and abnormal baseline blood potassium can be adjusted. Intraoperative blood pressure should be closely monitored to avoid the occurrence of hypotension, and the operation time should be shortened as much as possible to reduce the risk of AKI. In terms of disease monitoring and prognosis judgment, patients with risk factors should be closely monitored after surgery. Once abnormal renal function indicators are detected, combined with risk factors, AKI can be determined in time, and intervention measures can be implemented quickly to improve patient prognosis [52]. In addition, these risk factors can also help doctors evaluate the prognosis of patients, and provide a strong basis for subsequent rehabilitation treatment and follow-up plans.

### Limitations of this study

This study is the first to conduct a meta-analysis on the incidence and influencing factors of postoperative AKI in elderly patients with hip fractures both domestically and internationally. Sixteen newly published articles in recent years were also included, which has certain novelty. However, this study also has certain limitations: firstly, there are many influencing factors, and due to limited research literature, further meta-analysis cannot be conducted, such as bone cement surgery, use of anticoagulants or platelet drugs, dementia, semi hip replacement surgery, etc. Therefore, the results of meta-analysis may have some bias. Secondly, this study conducted a meta-analysis on the incidence of AKI and found significant heterogeneity, ultimately determining that this heterogeneity is caused by the duration of the study and sample size, which may have an impact on the results. Finally, there is a discrepancy between our research findings and those of Li et al [53]. In our study, postoperative albumin was not a contributing factor to acute kidney injury in elderly patients with hip fractures after surgery, which may be related to differences in the study population, study region, and total sample size. Therefore, in the future, multicenter, large-scale, and high-quality studies are still needed to comprehensively evaluate the influencing factors of postoperative AKI in elderly patients with hip fractures.

## Conclusion

This study included 22 articles for systematic analysis, and the results showed that the incidence rate of AKI in elderly patients with hip fracture after surgery was 17.2%. Baseline serum potassium, hypertension, chronic kidney disease, diabetes, operative hypotension and operative time are the influencing factors of AKI in elderly patients with hip fracture after surgery. This provides some basis for early identification of influencing factors and prevention of postoperative AKI in the future.

## Supporting information

S1 Table PRISMA checklist. (DOCX)

S2 Table Search strategy. (DOCX)

S1 Data. (XLSX)
