# Supplementary material for: The incidence and influencing factors of postoperative acute kidney injury in elderly patients with hip fractures: A meta-analyses
Source: PLoS One. 2025 Jun 20;20(6):e0322228. doi: 10.1371/journal.pone.0322228 (PMC12180726; doi:10.1371/journal.pone.0322228)
Supplement: S2 Table — (DOCX) [file pone.0322228.s002.docx]

**Table2 Search strategy**

| **Datebase** | **Search strategy** | **Total** |
| --- | --- | --- |
| PubMed | ((((((("Acute Kidney Injury"[Mesh]) OR (acute renal injury)) OR (acute renal insufficiency)) OR (acute renal failure)) OR (acute kidney failure)) OR (AKI)) AND (((((("Hip Fractures"[Mesh]) OR (hip,fracture)) OR (femoral neck fracture)) OR (intertrochanteric fracture)) OR (subtrochanteric fractures)) OR (trochanteric fractures))) AND (((((((("Risk Factors"[Mesh]) OR (Predicted factor*)) OR (Reason*)) OR (Correlated*)) OR (Predictor*)) OR (influen*)) OR (inciden*)) OR (risk,factors)) | 148 |
| Web Of Science | (Hip Fractures OR "hip,fracture" OR "femoral neck fracture" OR "intertrochanteric fracture" OR "subtrochanteric fractures" OR "trochanteric fractures" ) AND ("Acute Kidney Injury" OR "acute renal injury" OR "acute renal insufficiency" OR "acute renal failure" OR "acute kidney failure" OR AKI) AND (Risk Factors OR "Predicted factor*" OR Reason* OR Correlated* OR Predictor* OR influen* OR inciden* OR "risk,factors") | 84 |
| Embase | ('hip fractures'/exp OR 'hip fractures' OR 'hip,fracture'/exp OR 'hip,fracture' OR 'femoral neck fracture'/exp OR 'femoral neck fracture' OR 'intertrochanteric fracture'/exp OR 'intertrochanteric fracture' OR 'subtrochanteric fractures' OR 'trochanteric fractures') AND ('acute kidney injury'/exp OR 'acute kidney injury' OR 'acute renal injury'/exp OR 'acute renal injury' OR 'acute renal insufficiency'/exp OR 'acute renal insufficiency' OR 'acute renal failure'/exp OR 'acute renal failure' OR 'acute kidney failure'/exp OR 'acute kidney failure' OR aki) AND ('risk factors'/exp OR 'risk factors' OR 'predicted factor*' OR reason* OR correlated* OR predictor* OR influen* OR inciden* OR 'risk,factors'/exp OR 'risk,factors') | 329 |
| Cochrane Library | (Hip Fractures):ti,ab,kw OR (hip,fracture) :ti,ab,kw OR (femoral neck fracture) :ti,ab,kw OR (intertrochanteric fracture) :ti,ab,kw OR (subtrochanteric fractures) :ti,ab,kw OR (trochanteric fractures) :ti,ab,kw AND (Acute Kidney Injury) :ti,ab,kw OR (acute renal injury) :ti,ab,kw OR (acute renal insufficiency) :ti,ab,kw OR (acute renal failure) :ti,ab,kw OR (acute kidney failure) :ti,ab,kw OR (AKI) :ti,ab,kw AND (Risk Factors) :ti,ab,kw OR (Predicted factor*):ti,ab,kw OR (Reason*):ti,ab,kw OR (Correlated*):ti,ab,kw OR (Predictor*):ti,ab,kw OR (influen*):ti,ab,kw OR (inciden*):ti,ab,kw OR (risk,factors) :ti,ab,kw | 25 |
| China Knowledge Resource Integrated Database (CNKI) | (SU=髋部骨折 + 转子间骨折 + 转子下骨折 + 股骨颈骨折 + 髋关节骨折 + 粗隆下骨折 + 股骨粗隆间骨折) and (SU=肾衰竭 + 肾损伤 + AKI + 肾功能不全 + 肾功能损伤 + 肾损害) and (SU=预测因素 + 影响因素 + 危险因素 + 相关因素) | 9 |
| Wanfang Database | (主题：（髋部骨折 OR 转子间骨折 OR 转子下骨折 OR 股骨颈骨折 OR 髋关节骨折 OR 粗隆下骨折 OR 股骨粗隆间骨折） and（主题：肾衰竭 OR 肾损伤 OR AKI OR 肾功能不全 OR 肾功能损伤 OR 肾损害） and (主题：预测因素 OR 影响因素 OR 危险因素 OR 相关因素) | 49 |
| Chinese Biomedical Database (CBM) | (髋部骨折 OR 转子间骨折 OR 转子下骨折 OR 股骨颈骨折 OR 髋关节骨折 OR 粗隆下骨折 OR 股骨粗隆间骨折） and（肾衰竭 OR 肾损伤 OR AKI OR 肾功能不全 OR 肾功能损伤 OR 肾损害）and (预测因素 OR 影响因素 OR 危险因素 OR 相关因素) | 33 |
| Weipu Database (VIP) | (篇关摘：（髋部骨折 OR 转子间骨折 OR 转子下骨折 OR 股骨颈骨折 OR 髋关节骨折 OR 粗隆下骨折 OR 股骨粗隆间骨折） and（篇关摘：肾衰竭 OR 肾损伤 OR AKI OR 肾功能不全 OR 肾功能损伤 OR 肾损害） and (篇关摘：预测因素 OR 影响因素 OR 危险因素 OR 相关因素) | 4 |
